# Case Report: ST-Segment Elevation in a Man With Acute Pericarditis

**DOI:** 10.3389/fcvm.2020.609691

**Published:** 2020-12-23

**Authors:** Yi-Ming Li, Yu-Heng Jia, Jiay-Yu Tsauo, Si Wang, Yong Peng

**Affiliations:** Department of Cardiology, West China Hospital, Sichuan University, Chengdu, China

**Keywords:** purulent pericarditis, esophageal perforation, STEMI, ECG, acute pericarditis

## Abstract

**Background:** Acute pericarditis is a rapid inflammatory condition of the pericardium with both infectious and non-infectious etiology. Most acute pericarditis is self-limited, with a small portion evolving rapidly. The definitive diagnosis of acute pericarditis often requires detailed physical examination, ECG, echocardiography, blood analysis and chest X-ray. It's usually challenging to distinguish acute pericarditis from ST-elevated myocardial infarction (STEMI) due to the similar ECG characteristics (ST segment change). Here we present a case of purulent pericarditis probably caused by esophageal perforation.

**Case:** A 52 year-old male presented with chest pain and dyspnea for 16 h. ST-segment elevation and positive cardiac markers lead to the initial diagnosis of ST-elevated myocardial infarction. Coronary angiography demonstrated normal coronary artery, while transthoracic echocardiography (TTE) showed massive pericardial effusion. Then, pericardiocentesis was performed with 250 ml of yellowish-green pus-like fluid extracted. A detailed history examination revealed a week history of possible esophageal perforation caused by a fishbone. And a further computed tomography (CT) demonstrated the presence of pneumomediastinum, and effusions in mediastinum, which lead to the diagnosis of purulent pericarditis. However, the patient's family refused further treatment and the patient died soon after discharge.

**Conclusion:** The differential diagnosis of chest pain should include acute pericarditis, which can be equally critical and fatal. And it's important to note the peculiar characteristics of acute pericarditis, which include concave and diffused ST-segment elevation, PR segment depression, and the ratio of ST-segment elevation to T wave >0.24 in lead V6. Moreover, comprehensive medical history and physical examination are crucial to the differential diagnosis of chest pain patients.

## Introduction

To quickly and effectively identify high-risk chest pain individuals is of great clinical importance and essential in emergency medical practice. From the results of a large multi-center study, chest pain patients accounted for about 5% of all emergency visits (1), and acute myocardial infarction (AMI) only accounted for a quarter of them.

Acute pericarditis is an important cause of non-ischemic related chest pain with ST segment changes, which makes it challenging to be distinguished from AMI. There are various causes associated with it, both infectious (viral, bacterial) and non-infectious (auto-immune disease, cancerous, traumatic) (2). The definitive diagnosis often requires auscultation (pericardial rub), ECG, echocardiography, blood analysis and chest X-ray (2). Most acute pericarditis is self-limited with favorable prognosis, while a small portion of them—the purulent pericarditis—can be devastating. Here we present a case of purulent pericarditis caused by esophageal perforation.

## Case

**Figure 1:**
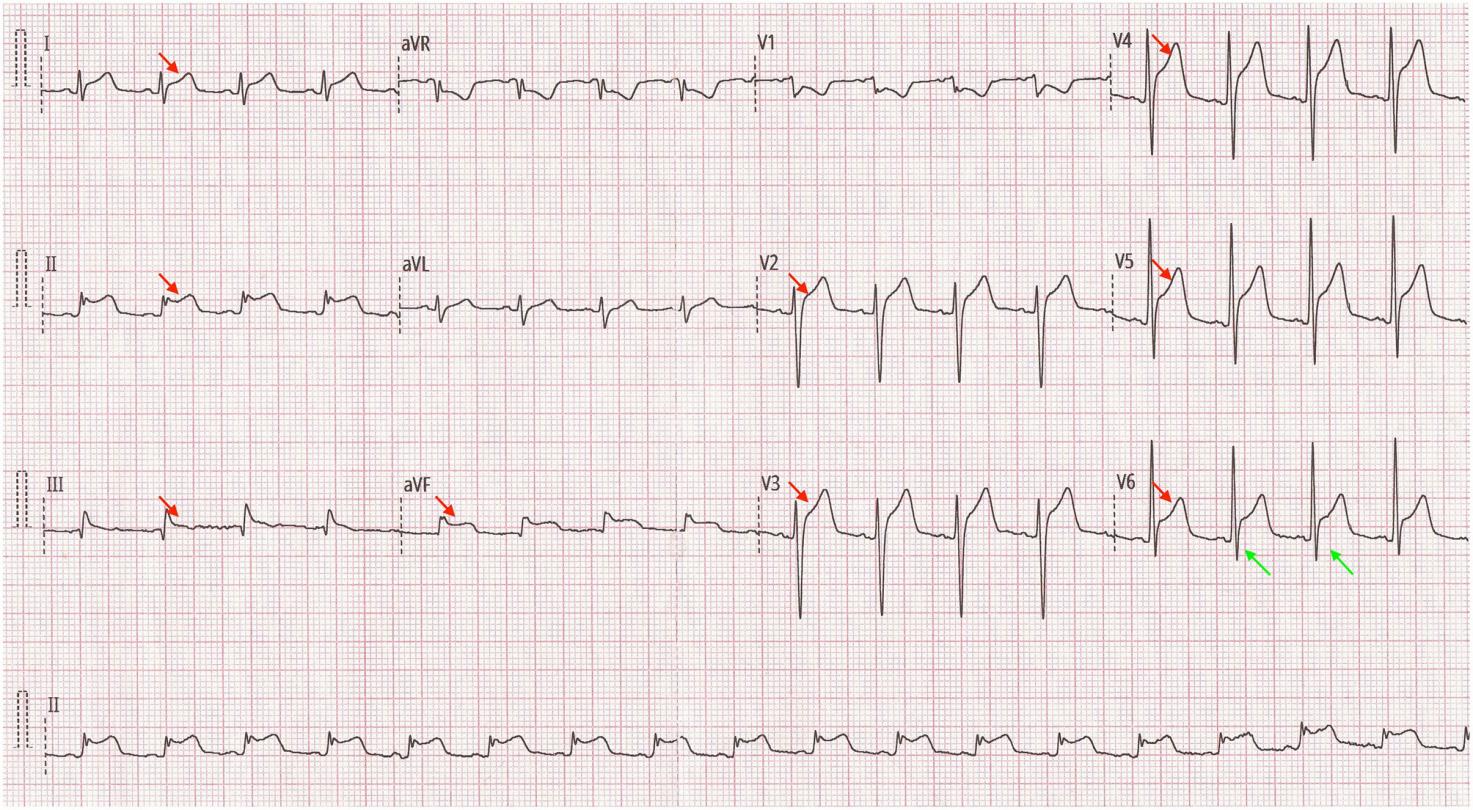
Twelve-lead electrocardiogram findings on admission (red arrows: concave ST-segment elevation at the extensive inferior and lateral leads; green arrows: ratio of ST-segment elevation to T wave >0.24 in lead V6).

A 52 year-old male with continuous chest pain and dyspnea for 16 h was admitted to our hospital. Physical examination was unremarkable, and vital signs were normal. His initial troponin-T level was 20.9 ng/L (limit of reference, 0–14), and the NT-proBNP level was 2,774 ng/L (0–227). Twelve-lead electrocardiogram (ECG) findings included ST-segment elevation at the inferior and lateral leads (I, II, III, AVF, and V2 to V6) (Figure 1).

**Figure 2:**
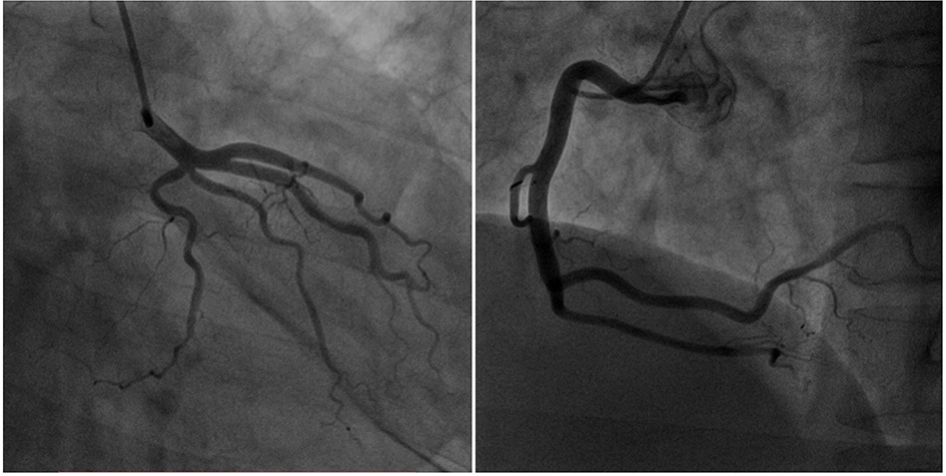
Emergency coronary angiography demonstrated normal patent left and right coronary arteries.

**Figure 3:**
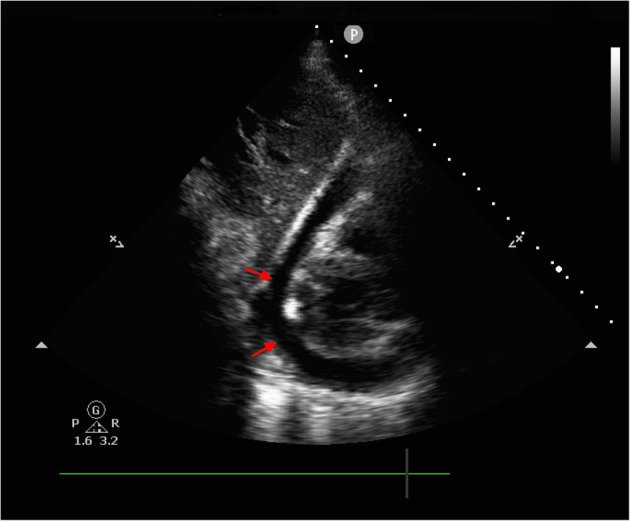
Bedside transthoracic echocardiography revealed massive pericardial effusion (red arrows: pericardial fluid sonolucent area).

Due to the typical presentation of chest pain, positive biomarkers and ECG findings, the initial diagnosis was ST-Elevation myocardial infarction (STEMI). The patient was immediately transferred for emergency coronary angiography (CAG), which demonstrated normal patent left and right coronary arteries (Figure 2). Next, a bedside transthoracic echocardiography (TTE) was performed and revealed a massive pericardial effusion (Figure 3). Furthermore, his blood routine examination showed leukocytosis (32.42 × 10^9^/*L*) and elevated neutrophil count (88.2%). Considering the possibilities of an acute pericarditis, a pericardiocentesis was performed. And about 250 ml of yellowish-green pus-like fluid was extracted, with biochemical examination revealed elevated lactic dehydrogenase (TLDH) (5,443 IU/L) and positive pus cells (Other biochemical results included 3,400 × 10^6^/*L* of nucleated cells, 1,000 × 10^6^/*L* of red cells, 52.5 g/L of total protein, 30.3 g/L of albumin, 146.0 IU/L of adenosine deaminase, <0.11 mmol/L of glucose, 133 mmol/L of sodium, 3.4 mmol/L of potassium and 93 mmol/L of chlorine). Therefore, purulent pericarditis was suspected. Consequently, a furthered detailed history exam was conducted, which then, the patient further revealed a week history of sore throat with prior fever as well as dysphagia for 4 days that self-resolved. Furthermore, the patient complained that a “fishbone got stuck in his throat more than a week ago.” Due to the combined presentation of chest pain, dyspnea, history of esophageal injury, and purulent pericardiocentesis fluid, we suspected a pathogenesis of esophageal perforation resulting in mediastinal infection leading to purulent pericarditis.

**Figure 4:**
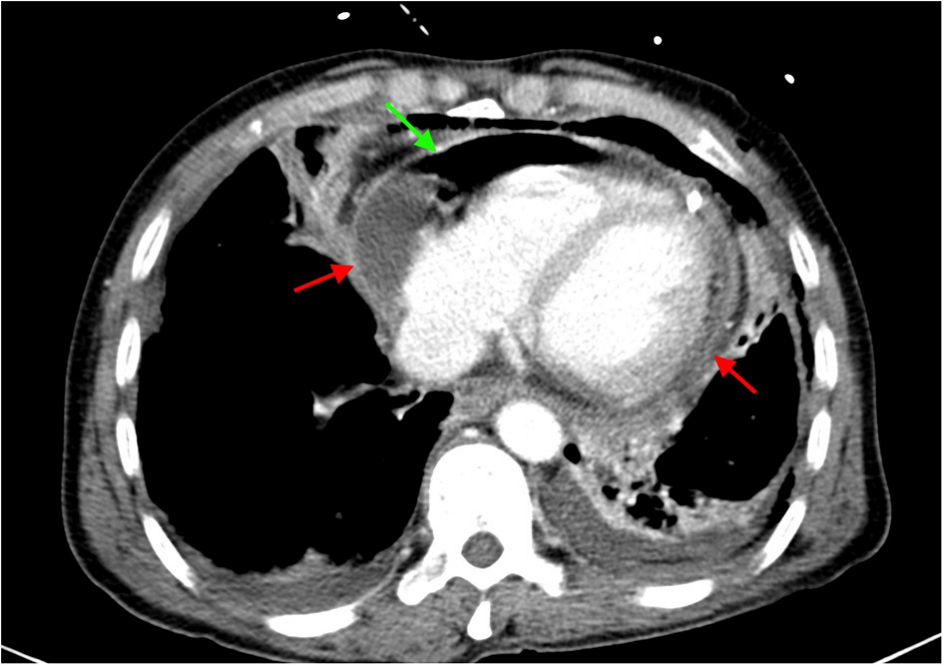
Computed tomography demonstrated enlarged heart with pneumopericardium and effusions in mediastinum and pericardium (green arrow: pneumopericardium; red arrow: effusions).

**Figure 5:**
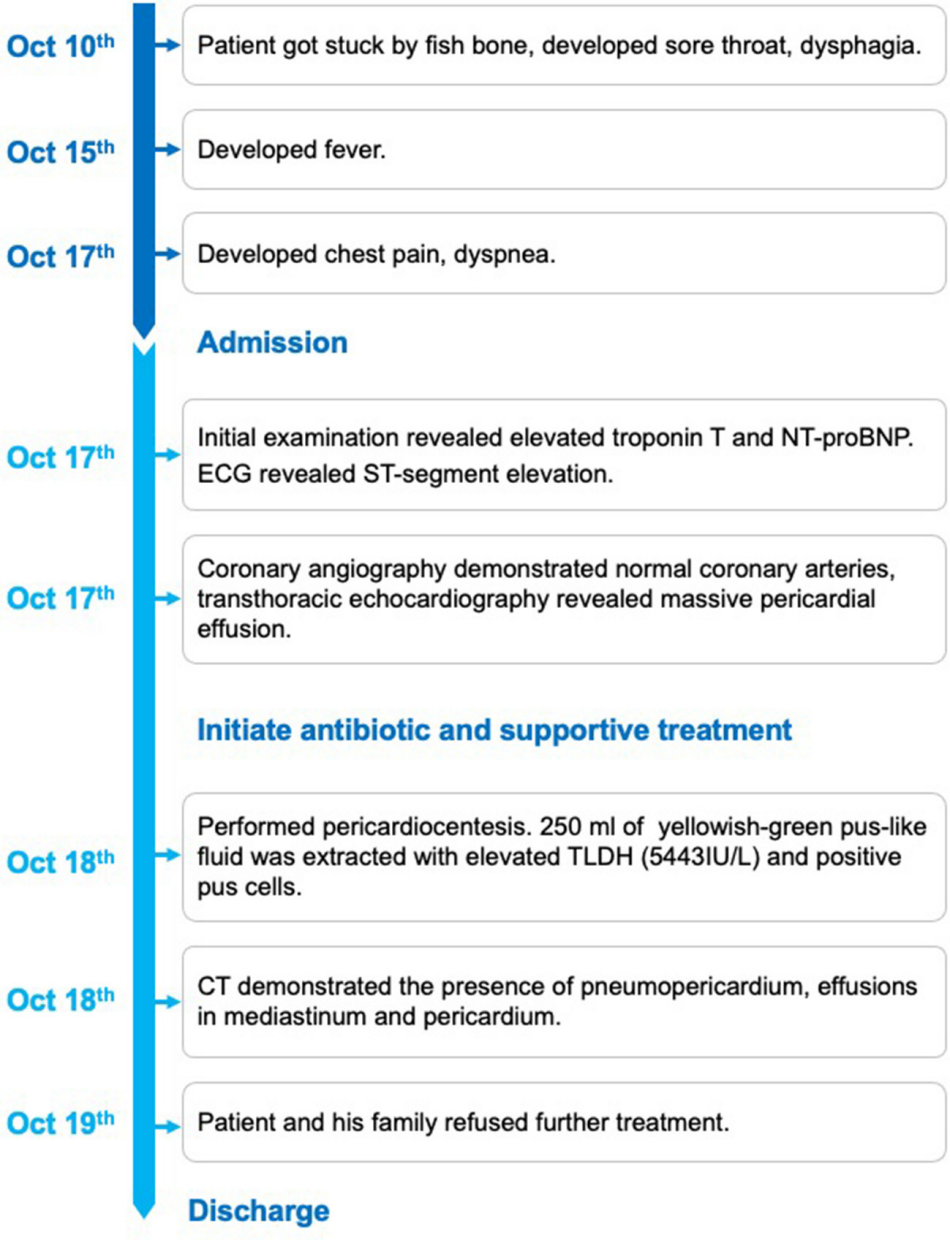
The detailed timeline of this patient from the symptoms onset to discharge and death.

Moreover, computed tomography (CT) further demonstrated the presence of pneumopericardium, effusions in mediastinum and pericardium (Figure 4). The heart team highly recommended immediate gastroscopy and surgery. However, the patient and his family refused further treatment considering the associated surgical risk and poor prognosis of this disease. We did not get to perform the cultivate of the puncture fluid either due to the requested discharge. The patient passed away shortly the next day after hospital discharge. We posted the detailed timeline of this patient from the symptoms onset to discharge and death (Figure 5).

## Timeline

### Discussion

Acute pericarditis is an important cause of non-ischemic related chest pain with ST segment changes. It is worthy to note that the characteristics of the chest pain is usually sharp, stabbing-like, and occasionally dull. Furthermore, the pain is often pleuritic and positional, and friction rub may occur in around 85% of the cases (3).

Compared to STEMI, patients with acute pericarditis often presents with ST-segment elevation and PR-segment depression, and around 80% of these patients may also have elevated troponin. Nevertheless, the ECG characteristics of ST-segment elevation in acute pericarditis is concaved and diffused. Whereas, the changes are concaved and localized in AMI and limited to leads III, AVF and V12 in pulmonary embolism. One of the specific ECG features of pericarditis is that the ratio of ST-segment elevation to T wave is >0.24 in lead V6 (which was approximately 0.4 in this case) (4). However, due to the similarities in presentation in pericarditis and ischemic-related chest pain, such cases still require coronary angiography to rule out coronary artery disease.

Purulent pericarditis with esophageal-pericardial fistula secondary to esophageal cancer was not rare from published reports. However, severe infection and bacterial-related pericardial effusion caused by esophageal perforation from an external object/body (in this case, a fishbone was the suspected culprit) is rare, and only few similar cases were ever published before (5). From the result of recent published literatures, the incidences of purulent pericarditis ranged from 0.7 to 1.0% of those with acute pericarditis in individual studies (6, 7). Although the occurrence rate seems low, but if occurred, the outcome is often devastating with a mortality rate of 20–40% even with active treatment (6, 8). Furthermore, repeated or prolonged infection may lead to fibrin deposition and pericardial adhesion, which is a significant negative prognosis factor. Therefore, vigorous antibiotic treatment should be immediately initiated in these cases, and if necessary, pericardiectomy (9). Additionally, some studies have also found that fibrinolysis may reduce the risk of constrictive pericarditis (10).

The biggest limitation in this study is that the suspected esophageal perforation was not confirmed by endoscope, surgery or autopsy. However, due to the patient's history and existing evidence, the heart team highly supported this diagnosis. In this case, we highlighted the importance of ECG analysis and an accurate history in the early differential diagnosis of life-threatening pericarditis.

## Data Availability Statement

The original contributions presented in the study are included in the article/supplementary material, further inquiries can be directed to the corresponding author/s.

## Ethics Statement

Written informed consent was obtained from the individual(s) for the publication of any potentially identifiable images or data included in this article.

## Author Contributions

All authors listed have made a substantial, direct and intellectual contribution to the work, and approved it for publication.

## Conflict of Interest

The authors declare that the research was conducted in the absence of any commercial or financial relationships that could be construed as a potential conflict of interest.

## References

[1] Emergency Department Patients With Chest Pain Writing Panal Rybicki FJ Udelson JE Peacock WF Goldhaber SZ Isselbacher EM 2015 ACR/ACC/AHA/AATS/ACEP/ASNC/NASCI/SAEM/SCCT/SCMR/SCPC/SNMMI/STR/STS appropriate utilization of cardiovascular imaging in Emergency Department patients with chest pain: a joint document of the American College of Radiology Appropriateness Criteria Committee and the American College of Cardiology Appropriate Use Criteria Task Force. J Am Coll Radiol. (2016) 13:e1–29. 10.1016/j.jacr.2015.07.007 26810814

[2] Maisch B Seferović PM Ristić AD Erbel R Rienmüller R Adler Y Guidelines on the diagnosis and management of pericardial diseases executive summary; The Task force on the diagnosis and management of pericardial diseases of the European society of cardiology. Eur Heart J. (2004) 25:587–610. 10.1016/j.ehj.2004.02.002 15120056

[3] Lange RA Hillis LD. Clinical practice. Acute pericarditis. N Engl J Med. (2004) 351:2195–202. 10.1056/NEJMcp041997 15548780

[4] Ginzton LE Laks MM. The differential diagnosis of acute pericarditis from the normal variant: new electrocardiographic criteria. Circulation. (1982) 65:1004–9. 10.1161/01.CIR.65.5.1004 7074735

[5] Chikuie E Fugisaki S Fukuhara S Imaoka K Hirata Y Fukuda S A rare case oesophageal perforation by a fish bone, leading to pericardial penetration and cardiac tamponade. Hiroshima J Med Sci. (2018) 67:47–9. 10.24811/hjms.67.2_47

[6] Sagristà-Sauleda J Barrabés JA Permanyer-Miralda G Soler-Soler J. Purulent pericarditis: review of a 20-year experience in a general hospital. J Am Coll Cardiol. (1993) 22:1661–5. 10.1016/0735-1097(93)90592-O 8227835

[7] Imazio M Cecchi E Demichelis B Ierna S Demarie D Ghisio A. Indicators of poor prognosis of acute pericarditis. Circulation. (2007) 115:2739–44. 10.1161/CIRCULATIONAHA.106.662114 17502574

[8] Pankuweit S Ristic AD Seferovic PM Maisch B. Bacterial pericarditis: diagnosis and management. Am J Cardiovasc Drugs. (2005) 5:103–12. 10.2165/00129784-200505020-00004 15725041

[9] Parikh SV Memon N Echols M Shah J McGuire DK Keeley EC. Purulent pericarditis: report of 2 cases and review of the literature. Medicine. (2009) 88:52–65. 10.1097/MD.0b013e318194432b 19352300

[10] Augustin P Desmard M Mordant P Lasocki S Maury JM Heming N. Clinical review: intrapericardial fibrinolysis in management of purulent pericarditis. Crit Care. (2011) 15:220. 10.1186/cc10022 21575282 PMC3219308

